# A promising research direction for colorectal cancer immunotherapy: The regulatory mechanism of CCL5 in colorectal cancer

**DOI:** 10.3389/fonc.2022.1020400

**Published:** 2022-11-01

**Authors:** Yuansen Li, Yi Lei, Jiaxue Sun, Wanfu Zhang, Xiaogang Li, Sijing Chen, Deshenyue Kong, Cheng Chen, Ke Bi, Xiao Luo, Hui Wang, Bo Li, Huayou Luo, Yu Xu

**Affiliations:** ^1^ Department of Gastrointestinal and Hernia Surgery, The First Affiliated Hospital of Kunming Medical University, Kunming, China; ^2^ National Health Commission (NHC) Key Laboratory of Drug Addiction Medicine, Kunming Medical University, Kunming, China; ^3^ Affiliated Hospital of Yunnan University, Kunming, Yunnan, China; ^4^ Yan’an Hospital of Kunming City, Kunming, Yunnan, China; ^5^ Yunnan Institute of Digestive Disease, The First Affiliated Hospital of Kunming Medical University, Kunming, Yunnan, China

**Keywords:** colorectal cancer, immunotherapy, chemokines, CCL5, the tumor microenvironment

## Abstract

Colorectal cancer (CRC) is one of the leading causes of cancer death worldwide, with high morbidity and mortality rates worldwide. Therefore, there is an urgent need to develop more effective treatments for CRC patients. In recent years, there has been some success in the immunotherapy of tumors, and immunotherapy has been used in many solid tumors including CRC. To date, the clinical efficacy of immunotherapy for CRC is limited, so more effective immunotherapy methods need to be explored. In patients with CRC, the CC chemokine CCL5 plays a role in the development of CRC and the recruitment and activation of immune cells, suggesting that it has potential for immunotherapy. This review mainly introduces the latest advances in the study of CCL5 acting as a marker of CRC and related mechanisms of immunotherapy, as well as the latest understanding of how CCL5 is involved in the invasion and development of CRC.

## Introduction

Colorectal cancer (CRC) is a prevalent and deadly disease that kills approximately 900,000 people worldwide each year (1). CRC is the fourth deadliest cancer in the world (1), although new treatment options have become available in recent years, the prognosis for patients with metastatic colorectal cancer (mCRC) remains poor, with a low 5-year survival rate (around 14%) (2). In conclusion, CRC is one of the most important diseases affecting public health worldwide. Although early screening has improved the survival rate of CRC patients in China, the majority of patients are still not diagnosed or treated in time, delaying the optimal treatment period. In addition, some of the early-stage patients also develop metastatic colon cancer during their treatment. Therefore, it is essential to find key treatments.

It is well known that tumors and their environment interact with each other, and the two may promote or antagonize each other. The environment in which tumor cells interact with their surroundings is the tumor microenvironment (TME) (3). The TME includes various cell types (e.g., immune cells, malignant cells, endothelial cells, and stromal cells) and signaling molecules (e.g., epidermal growth factor (EGF), transforming growth factor-β (TGF-β), fibroblast growth factor (FGF), and tumor necrosis factor-α (TNF-α)), as well as blood vessels and stroma. The complex interaction between tumor cells and extracellular metabolites promotes and maintains the homeostasis of TME (4). The further development of tumors cannot be separated from their complex interaction with various cells, especially cancer cells, and immune cells (5). Almost all tumors have immune cell infiltration, but the immune response is different (6). Several studies have shown that the type, density, and location of immune cells within CRC tissue can predict clinical outcomes and disease progression (7). In the TME, the proportion of CD3+ and CD8+ T cells presented in CRC patients correlates with the risk of recurrence and survival (8). Dendritic cells can further enhance the immune response by producing cytokines, such as interleukin 12 (IL-12), which is produced by macrophages and dendritic cells and promotes the differentiation and activation of CD8+ T cells and NK cells (9, 10). Increased amounts of CD8+ TIL in the TME can exert an anti-tumor function. It has been shown that the combination of anti-TGF-β and anti-PD-L1 monoclonal antibodies promotes a significant increase in CD8+ TIL in a mouse model of CRC, further exerting an anti-tumor effect (11). At the same time, cytokines in the TME are essential for regulating the function of various tumor cells and stromal cells (12, 13).

Chemokines, named for their cellular chemotactic effects, are small molecules (molecular weight about 8-10 kDa) of cytokine family proteins whose main functions are targeted transport of immune cells and promotion of lymphoid tissue development (14). Chemokine proteins are structurally tertiary structures with four conserved cysteine residues, which are further classified into four main types based on the positions of the first two cysteine residues: CC-chemokine, CXC-chemokine, C-chemokine and CX3C-chemokine (15). Regulation of immune cell recruitment by chemokine networks is a complex process. Different chemokines can recruit various subsets of T cells into the TME to exert different immune actions or directly target tumor and stromal cells (16–18). For example, CCL2, CCL3 and CCL5 induce monocytes to secrete matrix metalloproteinase 9 (MMP9) to further promote tumor invasion and metastasis (19); CCL2 affects tumor vascularization and tumor metastasis by targeting colon cancer vascular endothelial cells (20); CCL8 promotes invasion and metastasis in breast, pancreatic, ovarian and prostate cancers (14) but high level of CCL8 expression in gastric cancer is a positive prognostic factor (21). CXCL9 and CXCL10 are endogenous tumor angiogenesis inhibitors and high levels of CXCL9 and CXCL10 promote increasing numbers of tumor-infiltrating CD8+ T cells and reduce the level of cancer metastasis and improve survival rate of patients with ovarian or colon cancer (7, 22, 23). Recruitment of TH17 cells into the TME *via* the CCL20-CCR6 axis may inhibit tumor progression. TH17 do not mediate antitumor activity by direct interaction with tumor cells but by recruitment of CD8+ T cells, NK cells and dendritic cells (DCs) into the TME (24–27). However, recruitment of TH22 cells into the TME *via* the CCL20-CCR6 axis may promote tumorigenesis (28, 29). Thus, the regulatory pattern of chemokine expression in cancer tissues and the mechanism of recruitment to immune cells will further improve the knowledge of chemokines on tumor immunity and tumor development. In addition, chemokine networks regulating immune cell recruitment in combination with other immunotherapies to treat tumors could lead to the development of therapies targeting a broader group of cancer patients (30). This will effectively facilitate the development of CRC immunotherapy and provide a scientific basis for the development of immunotherapy regimens for each patient.

CCL5 is a member of the C-C chemokine subfamily, which also includes CCL3 and CCL4 (31). CCL5, which also binds to other G protein-coupled receptors such as CCR1 and CCR3 (32), binds to its receptor CCR5 with high affinity. CCL5 is associated with a variety of biological processes and its expression in a variety of different tumors has implications for the development of cancer. For example; tumor-associated macrophage (TAM)-derived CCL5 or the addition of CCL5 promotes prostate cancer metastasis and drug resistance through a STAT3-dependent epithelial-mesenchymal transition process and upregulation of the transcription factor Nanog (33). Traditional type 1 dendritic cells (cDC1s) are essential for antitumor immunity and their presence in the TME is associated with improved prognosis in cancer patients. CDC1 is recruited through the CCL5/CCR5 axis to infiltrate into tumor tissue and then exert anti-tumor effects (34). Earlier studies indicated that CCL5 plays an important role in CRC. The infiltration of CD8+ T cells into primary CRC sites was significantly increased in CCL5 knockout mice (35). However, tumor patients with high CCL5 protein expression in some CRC patients indicates a better prognosis. This suggests that the mechanism of CCL5 action in CRC needs to be further elucidated.

## Tumor immunotherapy: One of the most promising areas of research in the field of cancer therapy

The main way to treat cancer is to remove the primary tumor and metastatic tumor cells, while preventing tumor recurrence. However, this is a difficult process and in the complex TME can greatly limit tumor clearance and treatment (36). The human immune system can recognize tumor cells and remove them, while tumor cells can also evade or block the immune cells in some way, thus promoting the development of tumor. Immunotherapy of tumor is a treatment to repair the immune system blocked by tumor and restore the normal anti-tumor immune response of the body, so as to cure the tumor patients or inhibit the development of tumor. Immunotherapy for tumors generally includes; immune checkpoint inhibitors, therapeutic antibodies, tumor vaccines, Cytokines (37), and small molecule inhibitors.

Immunotherapy has attracted a great deal of attention in the last few years, and immunotherapy has shown initial success in some solid tumors. T-cell infiltration at the tumor site is associated with good prognosis (38) and beneficial outcomes for patients with tumors. In addition, drugs that enhance the killing action of T cells on tumor cells (ipilimumab (targeting cytotoxic T lymphocyte-associated antigen-4(CTLA4)), pembrolizumab and nivolumab (targeting PD1)) have been approved for the treatment of certain solid tumors (39, 40).

Immunotherapy has been gradually increasing in patients with colorectal cancer. High tumor mutation load generates new antigens that further induce an immune response by tumor-infiltrating lymphocytes (TIL) recruited to the tumor site (41). Based on studies within the last few years, relatively reliable data have been reported for Tremelimumab (42) (anti-CTLA4 immunoglobulin G2 (IgG2) antibody), Nivolumab (43–46) (anti-PD1 antibody), and Nivolumab in combination with Ipilimumab (47) in the treatment of patients with d MMR-MSI-H CRC (Exhibits high tumor mutational load and high number of tumor-infiltrating lymphocytes). Immunotherapy with these agents (nivolumab and pembrolizumab alone or in combination with ipilimumab) has been used in the second-line treatment of patients with d MMR-MSI-H CRC (48). Immunotherapy does not show a significant advantage in p MMR-MSI-L CRC (Exhibits Low tumor mutational load and low number of tumor-infiltrating lymphocytes) patients (the vast majority of CRC patients have p MMR-MSI-L tumors), which may be related to the low number of immune cells recruited to the tumor, and the high proportion of p MMR-MSI-L CRC patients among mCRC patients. Therefore, it is essential to find effective treatments for p MMR-MSI-L CRC patients. In short, immunotherapy has gradually emerged in the treatment of CRC and has played a significant role. However, current immunotherapy has many unresolved problems and cannot completely solve a series of problems in the treatment of CRC.

## Mechanism of action of chemokine CCL5 in colorectal cancer holds promise as a new immunotherapeutic target

CCL5 may also promote the survival, proliferation, and invasion of tumor cells through different mechanisms. In human solid tumors, CCL5 expressed by tumor cells and CXCL9 expressed by both macrophages and DCs are important for tumor infiltration by T cells, a process that also involves the identification of tumor antigens by T cells. CCL5 is regularly epigenetically silenced in tumor cells but can be reactivated by decitabine (DNA methyltransferase inhibitor) (49). Natural killer (NK) cells produce CCL5 and XCL1 to promote cDC1 accumulation in tumors. CCL5 and CXCL9 overexpression is associated with CD8+ T cell infiltration in solid tumors. The NK cell/chemokine functional axis determines the abundance of cDC1 in human melanoma, breast, lung, head and neck squamous cell carcinomas and has been shown to affect patient survival (50). Glycogen branching enzyme (GBE1) blockade promotes the secretion of CCL5 and CXCL10 and recruits CD8+ T lymphocytes to TME *via* the IFN-I/STING signaling pathway (51). The growing number of studies accompanying the interaction between CCL5 and immune cells suggests that the CCL5/immune cell axis may be a promising target for cancer immunotherapy to achieve tumor regression, including in CRC.

### Diagnostic significance of CCL5 levels in colorectal cancer

CCL5 plays a role in multiple cancer stages, including cancer cell proliferation, migration, invasion, angiogenesis and immune regulation (31, 52). It has been suggested that CCL5 can be used as a biomarker and predictor for the development of anti-cancer treatment strategies (53). CCL5 expression in lung adenocarcinoma cells has been reported to be a predictor of survival in a subset of patients and may be used as a prognostic factor in lung cancer (53). In addition, the significance and availability of CCL5 acting as a potential biomarker in the early diagnosis of CRC was demonstrated in another study (54). At early diagnosis, CRC is highly treatable; however, screening rates for CRC in the general population remain low (55). CCR5, CCL5, PDGF and EphA7 levels were measured in blood samples from 70 CRC patients (To measure the above biomarkers, blood was collected preoperatively from patients undergoing tumor resection and patients in stage IV before chemotherapy without surgical intervention. The histological type of all patients was adenocarcinoma.) and 40 healthy individuals using ELISA. This supports the idea that CCL5 is a potential biomarker for the diagnosis of colon cancer (54). However, this study also has the limitation of small number of subjects to perform CRC staging analysis. The small sample size and the lack of further analysis of other variables may not yield reliable results. However, their study also pointed to a higher rate of accurate diagnosis when used in combination with PDGF, EphA7, and CCL5.

In the innate immune system, STAT1 is an important player in the protection of the host against pathogens, and STAT1 regulates the expression of multiple immunoregulatory genes, including type I interferons (56). One study analyzed that the mRNA levels of STAT1 and CCL5 were significantly higher in CRC tissue specimens(the 65 samples of GSE29621) compared to normal colon tissue(**Table 1** NO.1) (57). And the upregulation of STAT1-CCL5 axis promotes the proliferation of colon cancer cells. To further investigate the role of CCL5 in CRC, another study reported that CCL5 protein expression and further Spearman correlation tests were performed in 195 CRC tissue samples and 162 normal colorectal tissue samples. The results showed that high levels of CCL5 expression were associated with increased risk of tumor budding (r = 0.583, P < 0.001), deep tumor invasion (r = 0.244, P = 0.001), lymph node metastasis (r = 0.237, P = 0.001), colorectal peri-intestinal nodal deposition (r = 0.198, P = 0.005) and advanced TNM stage (r = 0.256, P < 0.001) were positively correlated (58). The high correlation between their tumor budding and high CCL5 expression suggests that CCL5 may be a potential diagnostic marker and therapeutic target for CRC tumor budding. Also, the correlation between high CCL5 expression and advanced TNM stage suggests that high CCL5 expression and advanced CRC stage are highly correlated.

**Table 1:** The cell origin of CCL5, the cause of up- or down-regulation, cell lines and animal models are described in the articles of each study.

No.	the cellular source of CCL5	CCL5 upward/downward adjustment	Research significance	CRC cell line in the experiment (human)	Mouse CRC experiments	DOI
1	CRC cell lines	CCL5-neutralizing antibody	STAT1 and CCL5 levels may be valuable biomarkers for CRC screening	HCT-116(MSI)、SW480(MSS) and SW620(MSS)	\	doi: 10.21037/atm-20-4428
2	Tumor cells, epithelial cells, lymphocytes	Alcohol consumption leads to high CCL5 expression in cancer cells including also lymphocytes and epithelial cells within the tumor. Artificial addition of CCL5 and CCL5-neutralizing antibody. knockdown of tumor-derived CCL5.	Targeted manipulation of CCL5-induced autophagy and the AMPK signaling pathway that mediates this induction may be a means to prevent CRC cell migration, which would improve CRC treatment and prevention.	HT29(MSS) DLD-1(MSI)	\	doi: 10.1038/s41598-018-26856-w
3	Macrophages	poly (I:C) and LPS treatment resulted in increased CCL5 secretion from macrophages	Inhibition of PD-L1 stability in CRC cells by inactivating the CCL5-mediated p65/STAT3-CSN5-PD-L1 pathway shows potential for the treatment of CRC.	HT29 (MSS) HCT116(MSI)	Mouse (C57BL/6 CT26) CRC allograft model	doi: 10.1038/s41418-019-0460-0
4	\	Knockdown of CCL5 of tumor and host origin	CCL5 Deficiency Caused by Combining CCL5 Neutralizing Antibodies with Anti-PD-1 Antibodies May Prolong Survival of CRC Patients	\	Mouse (BALB/c CT26 and C57/B6 MC38 ) CRC allograft model	doi: 10.1038/s41419-018-0796-2
5	CRC cell lines	Knockdown of tumor-derived CCL5	Tumor cells regulate antitumor immune responses by secreting CCL5, which attracts Treg cells to the tumor microenvironment and enhances their ability to kill CD8+ T cells.	\	Mouse (C57BL/6 CT26) CRC allograft model	doi: 10.1158/0008-5472.CAN-11-2493
6	hMSCs	TNF-α-activated-hMSCs secrete high levels of CCL5	The CCL5/CCR1/β-catenin/Slug pathway is responsible for the tumor-promoting effect of TNF-α-activated hMSCs on colon cancer development. Strategies targeting MSCS-cancer cell crosstalk should provide a new approach to cancer therapy.	SW1116(MSS)	Mouse (Balb/C HT29) xenograft model of CRC	doi: 10.1038/cddis.2017.138
7	\	\	The anti-CCR5 treatment blocked MSC-induced tumor progression, suggesting that inhibition of MSC-CRC interactions may be an effective CRC treatment strategy and emphasizing the need for clinical trials of these agents.	HCT116	A mixture of HCT116 cells overexpressing CCR5 and MSCs was inoculated subcutaneously into immunodeficient mice (KSN/slc nude mice)	doi: 10.1038/s41419-019-1508-2
8	dMMR CRC cell lines	CCL5 and CXCL10 overexpression is driven by active endogenous type I IFN signaling in dMMR CRCs but can be exogenously induced in CIN CRCs(Direct result: MLH1 missing for dMMR CRC)	Some tumors with high TMB are unable to stimulate endogenous antitumor immune responses and are not responsive to suppressive therapy. This study identifies potential therapeutic targets for inducing T-cell infiltration into CIN CRC. Enhanced T-cell infiltration will increase the likelihood of TIL activation, even in tumors with few neoantigens.	orthotopic model with isogenic CRC cells differing in their MMR capacity	Direct in situ implantation of dMMR and CIN MC38 CRC cells into the colonic wall of WT C57BL/6 mice by non-invasive endoscopic injection	doi: 10.1084/jem.20210108
9	CRC tumor buds	CRC tumor buds overexpress and secrete CCL5. artificially added CCL5 (in vitro).	CCL5 may serve as a potential diagnostic marker and therapeutic target for CRC tumor germination.	HCT-8, SW620(MSS),RKO(MSI),LS174T(MSI)	HCT116 cells secreting large amounts of CCL5 were used to establish an in situ CRC xenograft mouse (BALB/c) model	doi: 10.1186/s13046-022-02300-w
10	\	Artificially added CCL5 (in vitro), CCL5-neutralizing antibody (in vivo)	Interfering with CCL5 signaling may be one way to control mCRC progression, either alone or in combination with MPDGFRβ-directed therapies.	HT29 (MSS)	Induction of subcutaneous and hepatic tumors in mice (BALB/c or SCID CT26 HT29) by abdominal or subhepatic capsule injection of cells. Induction of lung metastases in mice (BALB/c or SCID CT26 HT29) by intravenous tail injection of cells	doi: 10.1371/journal.pone.0028842
11	T Cell-Derived CCL5. CCR5 is mainly expressed by metastatic tumor cells	T Cell-Derived CCL5, Artificially added CCL5, CCL5-neutralizing antibody	The efficacy of CCR5 blockers alleviates this pro-tumor inflammatory microenvironment, particularly through their effects on tumor cells and tumor-associated macrophages.	Primary cells extracted from ascites or blood	No mouse model reproduces the complex immune microenvironment of late-stage pretreated metastatic CRC, so a human tumor explant model was used for functional testing	doi:https://doi.org/10.1016/j.ccell.2016.03.005

The correlation between tumor-related markers in blood and the efficacy of chemotherapy has also been one of the important directions of research (59). Regorafenib is an oral multikinase inhibitor used in the third or higher line of treatment for metastatic CRC. Genetic variants in the CCL5/CCR5 pathway predict severe hand-foot skin reactions in patients with mCRC treated with regorafenib (60). Another study reported that reductions in CCL5 levels and vascular endothelial growth factor A (VEGF-A) levels may serve as potential predictors of survival or treatment-specific toxicity in mCRC patients treated with regorafenib (61). Serum samples were collected from 54 patients before treatment initiation, on day 21, and before disease progression. CCL5 levels ≤ the criterion at baseline (59959 pg/ml) were associated with relative tumor shrinkage (P = 0.021), better progression-free survival (PFS) (P = 0.036), and overall survival. At day 21, decreased VEGF A levels were significantly associated with better progression-free survival (P = 0.021) (61). Similarly, this study was limited by the small size of the sample.

Although CCL5 has been shown to be altered in most CRC (especially in late TNM), its value in CRC diagnosis is not yet sufficient as it is correspondingly altered in tumors (62) outside of CRC and in some inflammatory diseases (63). Its value in the diagnosis of CRC is not quite reliable. First, the role of CCL5 in CRC staging (early/late-stage) may be different; second, high expression of CCL5 in blood and tumor sites, respectively, may have different diagnostic implications. Finally, the role of CCL5 in different consensus molecular subtypes of CRC (CMS) is also likely to be different. However, unlike these results. Gen Nishikawa et al. analyzed Survival curves of overall survival (OS), cancer-specific survival (CSS), and relapse-free survival (RFS) by (ELISA) measuring preoperative serum levels of CCL3, CCL4, and CCL5 in 114 patients with CRC (TNM stage 0, I, II:67 TNM stage III, IV:47). However, no correlation was found between CCL5 concentration and prognosis (64). We speculate that this may be due to the different CRC stages and CRC types in the collected sample populations. It would be better to further discuss the type and stage of CRC. Since all the results mentioned above are very likely to depend on the number of patients in the trial and the different tumor stages as well as the different techniques of CCL5 detection, different results may be obtained. We believe that high expression of CCL5 in tumor sites is more likely to be a representative marker. High expression of CCL5 in serum as a diagnostic marker still needs further study and it is preferred to be used in combination with other serum markers for accurate diagnosis.

### Regulation of CCL5 in promoting the development of colorectal cancer

Alcohol consumption promotes tumor progression and elevated serum CCL5 levels, and studies have reported that ethanol increases CCL5 secretion in two CRC cell lines, HT29 and DLD-1, and that CCL5 activates cellular autophagy *via* the AMPK pathway, further inducing tumor metastasis (65)(**Table 1** NO.2).

Infiltrating macrophages in tumor are important, and TAMs are associated with poor prognosis in the vast majority of cancers (66), and TAMs promote tumorigenesis and progression by facilitating immune evasion of tumors.

**Figure 1:**
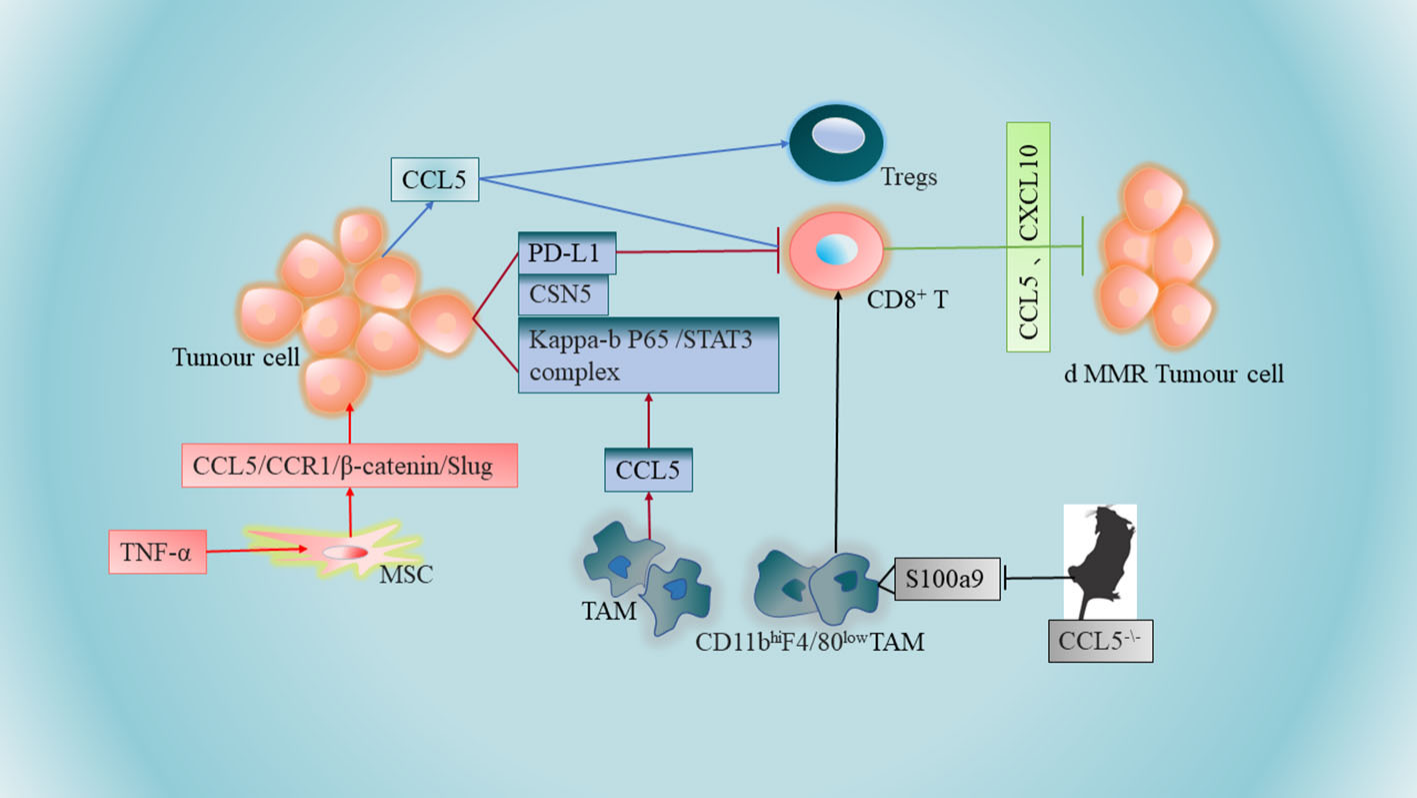
Tumor-associated macrophages (TAMs) promote immune escape by secreting CCL5. CCL5-deficient transgenic mice may further promote the aggregation of CD8+ T cells to tumor sites in CRC mouse models by inducing reduced expression of S100a9 (S100 calcium-binding protein A9) in CD11bhiF4/80 low tumor-associated macrophages. Higher levels of CCL5 expression in colon tumor cells promote apoptosis of CD8+ T cells and increase infiltration of regulatory T cells (Tregs). Increased secretion of CCL5 by TNF-α-stimulated mesenchymal stem cells (MSCs) can be accomplished by further activation of the epithelial-mesenchymal transition (EMT) process *via* the CCL5/CCR1/β-catenin/Slug pathway. Overexpression of chemokines CCL5 and CXCL10 in CRC cells lacking DNA mismatch repair (d MMR) recruited and activated systemic CD8+ T cells further selectively to enter and further exert anti-tumor immune effects.

It has been shown that macrophages promote immune escape by secreting CCL5. The mechanisms involved implies that CCL5 promotes the formation of the kappa-B p65/STAT3 complex, which activates COP9 signalosome 5 (CSN5) transcription, and then CSN5 further regulates the deubiquitination and stability of PD-L1, further inhibiting CD8+ T cell responses and thus leading to immune escape (67)(**Figure 1**). TAM-generated CCL5 induced a significant increase in PD-L1 expression and its ability to bind PD-1 in CRC cells, independent of the microsatellite status in HT29 (MSS) and HCT116 (MSI) cells. Thus, novel pathways of PD-L1 upregulation may facilitate the treatment of CRC, especially MSS CRC (**Table 1** NO.3). Another study suggests that CCL5-deficient transgenic mice may delay tumor growth and metastasis by inducing reduced expression of S100a9 (S100 calcium-binding protein A9) in CD11b^hi^F4/80^low^ tumor-associated macrophages, further promoting the accumulation of CD8+ T cells to tumor sites in a CRC mouse model (35)(**Figure 1**). In addition, CCL5 deficiency upregulates PD-1 and PD-L1 expression and reduces resistance to anti-PD-1 antibody therapy in a mouse model of CRC (35)(**Table 1** NO.4), The contradiction between PD-L1 expression and the above results may be due to *in vivo* experiments and the presence of more influential variables in CCL5-deficient transgenic mice, so more in-depth studies will be warranted to elucidate this discrepancy.

The lack of CD8+ T cells in the central tumor region of solid tumors has become a major barrier to immunotherapy, especially for patients with CRC. It has been shown that higher levels of CCL5 expression in human and mouse colon tumor cells promote apoptosis of CD8+ T cells and increase infiltration of regulatory T cells (Tregs). At the same time, CCL5 enhances Treg cell-mediated apoptosis of CD8+ T cells in a TGF-b-dependent manner, further playing a role in immune escape from CRC (52)(**Figure 1**).

Mesenchymal stem cells (MSCs) are one of the major components of the tumor stroma, and MSCs are an important component of CRC progression, conferring a transition to an aggressive and metastatic phenotype. Mesenchymal stem cells can promote CRC progression through paracrine neuromodulin 1 (NRG1)/HER3 signaling (68). It has been reported that increased CCL5 secretion by tumor necrosis factor (TNF)-α stimulated MSCs and then MSCs promote CRC cell proliferation and progression can be accomplished by further activation of the epithelial-mesenchymal transition (EMT) process through the CCL5/CCR1/β-catenin/Slug pathway (69)(**Figure 1**). This study also revealed a novel pathway by which MSCs promote colon cancer proliferation, migration and invasion, and highlighted the importance of CCL5 in the interaction between MSCs and cancer cells (**Table 1** NO.6). Notably, there are similarities compared to the role of CCL5 in breast cancer, whose cancer cells can stimulate MSCs to secrete CCL5, and in turn CCL5 promotes cancer cell invasion and metastasis (70). Meanwhile, it has been found that CCR5 is produced in large quantities in human MSCs, and the CCL3/4/5-CCR5 axis promotes tumor progression through the interaction between MSCs and CRC cells, suggesting that CCL3/4/5 secreted by MSCs may be an important factor in the interaction between MSCs and colorectal cancer cells in TME (64)(**Table 1** NO.7). CCR5 is usually absent on tumor cells in early primary CRC tumors (71). However, strong CCR5 expression was detected in the cytoplasm of several CRC cell lines (SW480, SW620, HT29, HCT116 and DLD-1) (64). This strongly suggests that the regulatory mechanism remains to be elucidated.

### Regulation of CCL5 in the anti-colorectal cancer

The presence of activated CD8+ T cells at the tumor site is an important positive prognostic marker for clinical response to immune checkpoint inhibitors in CRC (72, 73). IFN signaling regulates the production of chemokines, including CCL5, which then recruit T cells to the TME for immune modulation (74). CD8+ T cells develop into cytotoxic T lymphocytes (CTL) and eliminate tumor cells by releasing cytotoxic mediators, such as granzyme B (GzmB) and granulysin (Gnly). The results of the study used to analyze new methods for secretory immune mediators show that (1) early-stage tumors secrete more IFN-γ compared to advanced-stage tumors, (2) CRC with more type 1 T-cell activity secrete more CXCL10 and CCL5 (type 1 T-cell activity is important for prolonging patient survival (7)), (3) GzmB+ CD8+ T cells are associated with chemokines CXCL10 and CCL5 were positively correlated (75).

dMMR CRC is rich in CD8+ tumor-infiltrating lymphatic cells, and CD8+ T cells respond to a large number of neoantigens from their unstable genome. Results from the TCGA PanCancer Atlas database indicate that the expression of CCL5 and CXCL10 in dMMR CRC is higher than their chromosomal instability (CIN) CRC (76). Overexpression of IFN-dependent chemokines CCL5 and CXCL10 in CRC cells lacking DNA mismatch repair (d MMR) recruits and activates CD8+ T cells further selectively throughout the body into CRC lacking DNA mismatch repair to further exert anti-tumor immune effects (76)(**Figure 1**), which are key to the anti-tumor response (**Table 1** NO.8). The expression of chemokine receptors CCR5 and CXCR3 (the receptors for CCL5 and CXCL10) on CD8+ TIL was also significantly higher in dMMR compared with CIN CRC.

The CRC cell line used by Mowat et al (76) was the d MMR cell subtype, and secondly, the experimental mouse model of Mowat et al. was direct *in situ* implantation of dMMR and CIN MC38 CRC cells into the colonic wall of WT C57BL/6 mice, an experimental approach that is different from the transplantation tumor model, which is more relevant in terms of the research value of the model (**Table 1** NO.8). This also demonstrates that CCL5 is antitumorigenic in some specific patients (e.g., patients with d MMR CRC) but not pro-tumorigenic in all patients. This aptly demonstrates that successful tumor immunization is required for the coordination of multiple processes. Understanding the mechanisms of non-antigen-dependent immune regulation of CRC in TME can further facilitate the development of tumor immunotherapy.

Most studies have concluded that CCL5 promotes tumor progression, but its antitumor effects are evident in specific tumor microenvironments, particularly in TME infiltrated by large numbers of CD8+ T cells. CMS1 CRC is more inclined to high expression of CCL5 compared to other consensus molecular subtypes (76), and the TME of CMS1 CRC is heavily infiltrated and activates infiltrating immune cells (CD8+ T, CD40 ligand, helper T cells, NK). This also suggests that the antitumor immune effect of immune cells is more likely to be promoted when more immune cells infiltrate the CRC microenvironment and when the expression of CCL5 is higher.

### CCL5 and metastatic colorectal cancer

**Figure 2:**
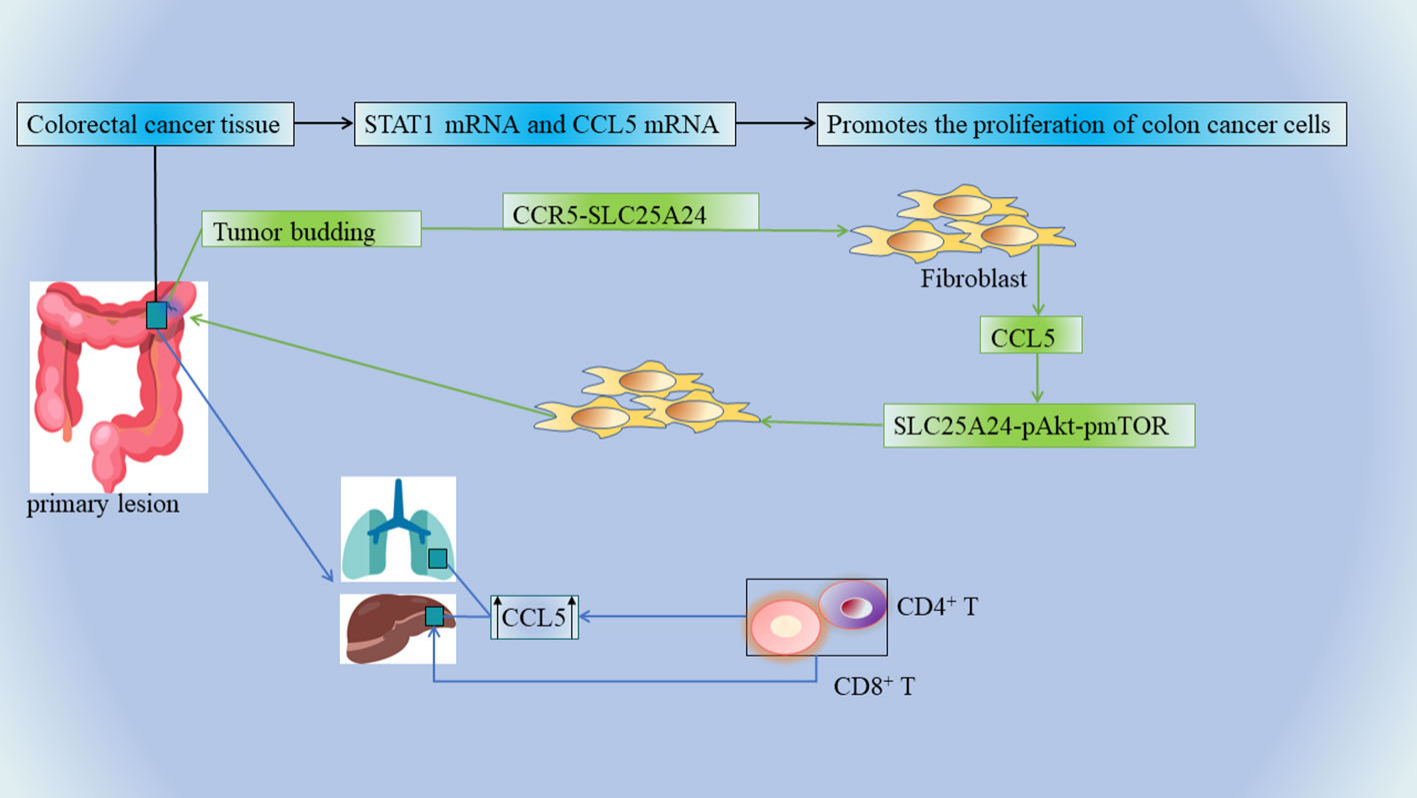
The mRNA levels of STAT1 and CCL5 were significantly elevated in CRC tissue specimens, and the upregulation of STAT1-CCL5 axis promoted the proliferation of CRC cells. Tumor buds, CCL5, and fibroblasts further promote angiogenesis and collagen synthesis, ultimately creating a tumor-friendly microenvironment. The invasive margins of CRC metastases contain multiple immune cells, and CCL5 is overexpressed in primary as well as liver and lung metastases. Chemokines recruit CD4+ and CD8+ T lymphocytes into the microenvironment, and infiltrating CD4+ and CD8+ T lymphocytes produce CCL5, which has multiple pro-tumor effects on the regulation of tumor cells and tumor-associated macrophages.

Tumor buds consisting of the most aggressive subpopulations of tumor cells are one of the tumor prognostic factors and they play a dominant role in tumor invasion (77). There is a close link between tumor outgrowth and promotion of tumor invasion in CRC, which is closely related to its unique immunosuppressive microenvironment. Tumor cells in CRC can recruit fibroblasts into tumor buds (58). There is a strong association between the number of tumor-associated fibroblasts (CAFs) and poor clinical outcomes in a variety of cancers, including breast, cervical, lung (78), bile duct (79), and CRC (80). Tumor bud-derived CCL5 can recruit fibroblasts *via* the CCR5-SLC25A24 signaling pathway, while fibroblasts are also considered to be an important source of CCL5 (81, 82). CCL5 also recruits fibroblasts *via* the SLC25A24-pAkt-pmTOR signaling pathway in fibroblasts, which further promotes angiogenesis and collagen synthesis through recruited fibroblasts, ultimately creating a pro-tumor microenvironment (58) (**Figure 2**). Therefore, CCL5 can be used as a potential diagnostic marker and therapeutic target for CRC tumor outgrowth (**Table 1** NO.9).

In CRC, unresectable tumor metastases (eg: liver metastases) portend a poor prognosis, and cancer patients usually die from tumor progression and metastatic burden (83, 84). The invasive margins of CRC metastases contain multiple immune cells, and such marginal areas are targets for chemotherapy. A team of researchers examined a large number of clinical specimens of human mCRC and found that CCL5 and its receptors were overexpressed in primary as well as liver and lung metastases (85)(**Figure 2**) (**Table 1** NO.10). This is also consistent with the above result of high expression of CCL5 in the advanced TNM stage (58). CCL5 secreted by lymphocytes promotes the proliferation of CRC cells capable of expressing CCR5, thereby promoting tumor growth in liver metastatic lesions (86). In mCRC, the immune microenvironment in the invasive margins (Non-central area) of CRC liver metastases whose chemokines recruit CD4+ and CD8+ T lymphocytes into the microenvironment, and infiltrating CD4+ and CD8+ T lymphocytes produce CCL5 has pleiotropic pro-tumor effects on the regulation of tumor cells and tumor-associated macrophages (87)(**Figure 2**). In this context, recent analysis of the CCL5-CCR5 axis in mCRC has revealed a new therapeutic option. Mainly because CCL5-CCR5 binding exerts tumor-stimulating effects (e.g., proliferation and production of tumor inflammatory cytokines) in mCRC, it is possible to antagonize metastatic tumors by means of CCR5 blockade (**Table 1** NO.11). In this study, a pilot clinical trial (MARACON) in patients with advanced metastatic colon cancer who had failed standard chemotherapy, CCR5 blocker treatment had no significant side effects and some patients achieved partial remission (87).

Previously reported randomized trials have clearly demonstrated a clear clinical impact of first-line chemotherapy in combination with anti-epidermal growth factor receptor (EGFR) or anti-vascular endothelial growth factor (VEGF) antibodies in the treatment regimen for mCRC (88, 89). Genetic variation in single nucleotide polymorphisms (SNPs) of CCL5 and CCR5 in patients with mCRC predicts the efficacy of the anti-epidermal growth factor receptor based on the location of the tumor (90). The CCL5/CCR5 axis activates protein kinase Cd (PKCδ), c-Src, and hypoxia-inducible factor-1a (HIF-1α) to regulate vascular endothelial growth factor production in a CCR5-dependent manner (91). Genotypes in the CCL5/CCR5 gene can identify specific populations that would benefit from bevacizumab (BEV: the first anti-angiogenic agent targeting VEGF-A that has been widely used in a variety of cancer types, including mCRC) in the first-line treatment of patients with mCRC.

CCL5 has been progressively investigated in tumor immunotherapy in recent years with some achievements. CCL5 plays a role in the recruitment and activation of immune cells, which means that CCL5 can be used as an adjuvant to enhance anti-tumor immunity through various protocols (92). An innovative single-domain antibody that bispecifically binds and neutralizes CCL2 and CCL5 (BisCCL2/5i) with high potency and specificity reverses the immunosuppressive process by which CCL2 and CCL5 attract Tumor-associated macrophages (TAMs) to infiltrate and induce their polarization toward the pro-tumor M2 phenotype. BisCCL2/5i promotes TAMs polarization toward the antitumor M1 phenotype and reduces immunosuppression in TME (93). The combination of BisCCL2/5i with a PD-1 ligand inhibitor (PD-Li) achieved long-term survival in mouse models of primary liver cancer and liver metastases from colorectal and pancreatic cancer (93). Another research indicates that immunotherapy combined with blockade of PD-L1 and CCL-5 may provide an effective treatment for patients with pancreatic ductal adenocarcinomas (PDAC) with high cancer Forkhead box protein 3 (Cancer-FOXP3 or C-FOXP3) levels (94). This effective chemokine-targeted therapeutic strategy could extend immunotherapy to a variety of human malignancies.

## Conclusion

There are numerous findings on the role of CCL5 in CRC development, with some reports suggesting that CCL5 inhibits tumor growth, while others suggesting that CCL5 promotes CRC development. The seemingly contradictory findings suggest that the detailed mechanism by which CCL5 acquires its tumor suppressor/promoter function in CRC development is largely unknown. The role played by CCL5 as an immunomodulatory factor is also complex and difficult to elucidate. Further studies are necessary to dissect the exact contribution of various factors, such as the role of CCL5 in the tumor microenvironment or immune system, and most studies have shown that modulation of CCL5 signaling appears to be an effective approach to provide therapeutic options against CRC. In addition, while the presence of specific subtypes of immune cells is beneficial to patients, cancer cells can also alter the immune microenvironment and immune cell function, leading to immunosuppression and immune evasion (95, 96). Due to the diversity of tumor-promoting cell types within CRC, current medical techniques need to achieve precise modulation of CCL5 expression in a particular cell type (**Table 1**), requiring treatments that combine multiple inhibitory measures targeting both cancer and stromal cells to produce effective tumor treatments.

In different stages of tumor development, its further in-depth study is more meaningful. Because most of the studies did not discuss CRC developmental stages separately in detail, and this also provides a new direction for our future research that CCL5 may play opposite roles in different developmental stages of CRC (7, 58)(**Table 1**).

In recent years despite advances in immunotherapy in CRC, CRC remains the leading cause of cancer-related deaths worldwide. The human immune system is highly correlated with the development and metastasis of CRC, and many studies have identified antagonistic or pro-tumorigenic effects of infiltrating immune cells. In these past years, therapeutic strategies that utilize the immune system to target cancer are rapidly evolving, such as the use of checkpoint inhibitors. Recently, immunotherapy in patients with d MMR-MSI-H CRC has shown significant and durable responses. However, the vast majority of CRCs are in p MMR-MSI-L CRC patients and are resistant to these inhibitors. The TME is closely related to immunotherapy, and clarifying the relationship between the TME and immune regulation would be a major advance in the treatment of CRC.

## Author contributions

YSL, DK, CC, JS, YX, LB, YL, HW, SC, XL, KB and HL conceived and designed the manuscript and prepared the manuscript. YSL, DK, JS, LB, YX, CC, YL, SC, XL, KB and HL performed the literature search. YSL, JS, YX, LB, DK, CC, YL, HW, SC, and HL did the picture making.YSL, WZ, LB, XL, YX, JS, HW, SC, XL, KB and HL revised the manuscript. All authors read and approve the final version of the manuscript.

## Funding

This work was supported by the Science and Technology Department of National Natural Science Foundation of China (82060525, NO.81860100), NO.202002AA100007, Yunnan Province (202001AS070004), NO.2019PT310003, NO.202101AY070001 -126, NO.2020DAMARA-005, Yunnan Province Intelligent Talent Platform (RLMY20200019), and Yunnan Provincial Health and Health Commission (202005AF150090), NO.YNWR-QNBJ-2019-243, NO.202005AC160057, NO.H-2018062.

## Conflict of interest

The authors declare that the research was conducted in the absence of any commercial or financial relationships that could be construed as a potential conflict of interest.

## Publisher’s note

All claims expressed in this article are solely those of the authors and do not necessarily represent those of their affiliated organizations, or those of the publisher, the editors and the reviewers. Any product that may be evaluated in this article, or claim that may be made by its manufacturer, is not guaranteed or endorsed by the publisher.
